# Phytochemicals and potential health effects of *Sambucus williamsii* Hance (*Jiegumu*)

**DOI:** 10.1186/s13020-016-0106-9

**Published:** 2016-07-28

**Authors:** Hui-Hui Xiao, Yan Zhang, Raymond Cooper, Xin-Sheng Yao, Man-Sau Wong

**Affiliations:** 1 State Key Laboratory of Chinese Medicine and Molecular Pharmacology (Incubation), Shenzhen Research Institute of The Hong Kong Polytechnic University, Shenzhen, 518057 China; 2 Department of Applied Biology and Chemical Technology, The Hong Kong Polytechnic University, Hung Hom, Kowloon, Hong Kong, China; 3 Spine Research Institute, Longhua Hospital, Shanghai University of Traditional Chinese Medicine, Shanghai, 200032 China; 4 Institute of Traditional Chinese Medicine & Natural Products, College of Pharmacy, Jinan University, Guangzhou, 510632 China

## Abstract

*Sambucus williamsii* Hance (*Jiegumu*) is traditionally used in Chinese medicine to treat bone and joint diseases. The major phytochemicals in *S. williamsii* are lignans, terpenoids, and phenolic acids, together with trace amounts of essential oils, minerals, amino acids, and natural pigments. In this review, a database search for studies published from 1990 to November 2015 was conducted using PubMed, the China Academic Journals Full-Text Database, and Google Scholar with the keywords “*Sambucus williamsii* Hance”, “*Sambucus williamsii*”, “*Sambucuswilliamsii* + clinic”, “*Sambucuswilliamsii* + biology”, “*Sambucuswilliamsii* + chemicals”, and “*Jiegumu*”, which covered chemical studies, cell culture studies, animal experiments, and clinical studies. This article reviewed the compounds isolated from *S. williamsii* that may reduce the risk of cancer, and exert antifungal, antioxidant, anti-inflammatory, bone fracture healing, and antiosteoporotic effects.

**Electronic supplementary material:**

The online version of this article (doi:10.1186/s13020-016-0106-9) contains supplementary material, which is available to authorized users.

## Background

*Sambucus williamsii* Hance (*Jiegumu*) is traditionally used in Chinese medicine to treat bone fractures, rheumatoid arthritis, gout, Kaschin–Beck disease, inflammation-related gastrointestinal disorders, kidney diseases, and wounds [1]. Recent studies [2–12] identified the phytochemicals in *S. williamsii* that exhibit various biological activities, including antifungal effects [2, 3], effects on the proliferation and differentiation of osteoblastic cells [4, 5], fracture healing effects [6], antioxidant, antiglycemic, and hypolipidemic activities [7], anti-inflammatory, gastroprotective, and antinociceptive properties [8, 9], and antiviral [10], antidiabetic [11], antimalarial [12], and antitumor [10] activities. This review describes these phytochemicals and their potential health benefits.

### Search strategy

**Fig. 1:**
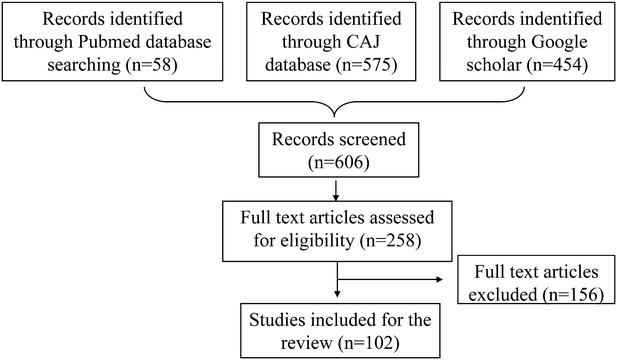
Flowchart of the search strategy used in this review

A database search for studies published from 1990 to November 2015 was conducted using PubMed, the China Academic Journals Full-Text Database, and Google Scholar with the keywords “*Sambucus williamsii* Hance”, “*Sambucus williamsii*”, “*Sambucuswilliamsii* + clinic”, “*Sambucuswilliamsii* + biology”, “*Sambucuswilliamsii* + chemicals”, and “Jiegumu”, which covered chemical studies, cell culture studies, animal experiments, and clinical studies. The latest paper was published in October 2015, and the full literature search is outlined in Fig. 1. Using the key terms described above, 1087 publications were found without limiting language, type, or content. All the hits were de-duplicated, and after restricting to English and Chinese languages, research articles, books, or theses, and titles or abstracts containing “*Sambucus williamsii* Hance” or “Jiegumu”, 606 papers were identified. Of these, 258 publications with full text were further extracted on the basis that they described chemical studies, in vitro activities, in vivo experiments, and clinical studies (omitting quantitative experiments, extraction and preparation methods, pharmacodynamic studies, and resource investigations). Finally, 102 papers were included in this review.

### Botanical characteristics

The genus *Sambucus* was originally placed in the family *Caprifoliaceae*, but subsequently reclassified to *Adoxaceae* according to genetic evidence and morphological comparisons, based on nucleotide sequences of the internally transcribed spacer region of nuclear ribosomal DNA, preliminary morphology, and a combination of the two data sets [13]. The family was reported to comprise at least 115 species and a large number of subspecific taxa [14, 15]. However, a recent revision by Bolli [16] recognized only nine species, with the remainder being synonymized or reduced to subspecific ranks. In China, there are five naturally occurring species within the *SambucusLinn.* genus: *S. williamsii* and its varieties *var. williamsii* and *var. miquelii* (*Nakai*), *Sambucus adnataWall.* (*Xuemancao*), *Sambucus sibiricaNakai* (*Xiboliya Jiegumu*) and *Sambucus chinensis Lindl.* (*Jiegucao*); and one introduced variety, *Sambucus nigra Linn.* (*Xiyang Jiegumu*) [17].

**Fig. 2:**
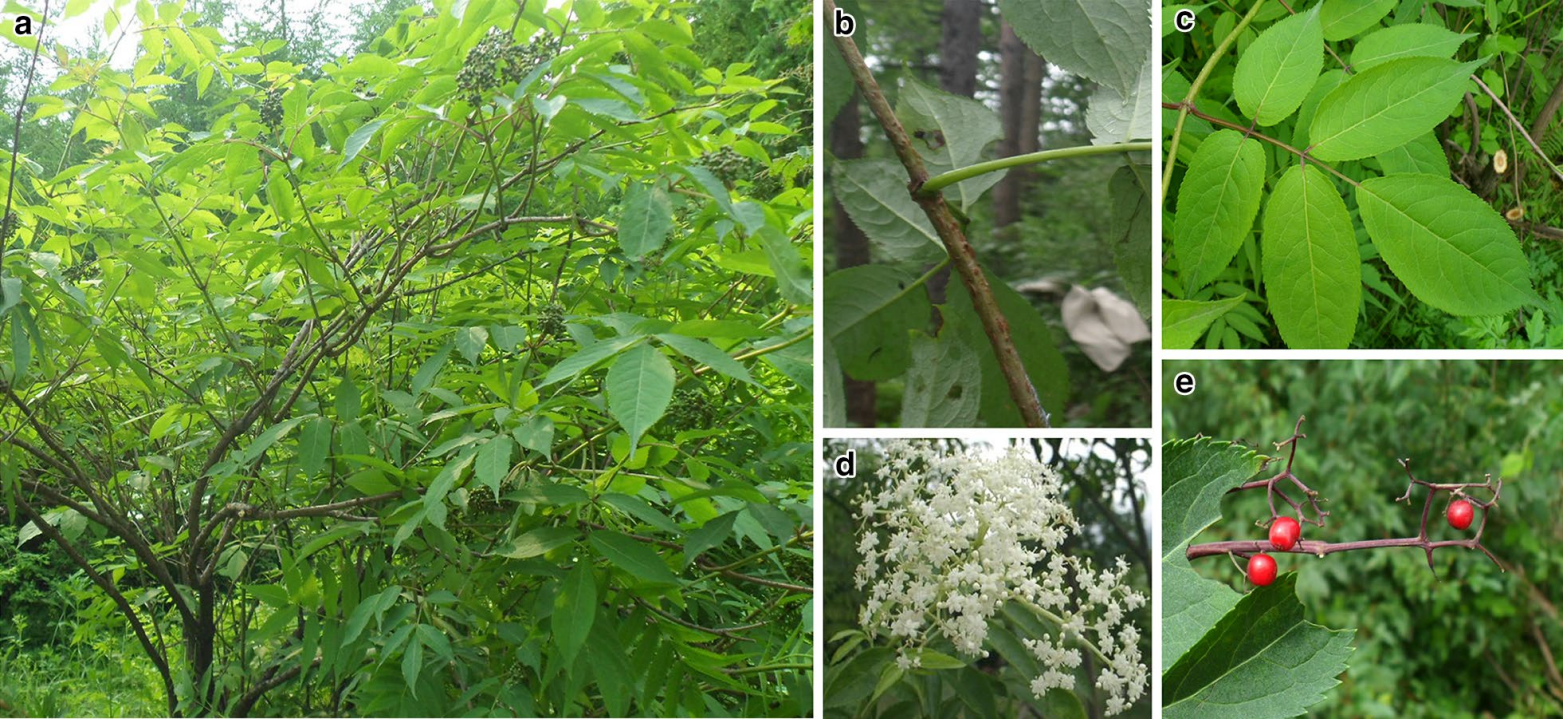
*S. williamsii* Hance (*Jiegumu*) is characterized by elliptic lenticels on branches, imparipinnate leaves with irregularly serrate margins, white or yellowish flowers, and small glossy red berries. **a** *S. williamsii* Hance; **b** branch; **c** leaf; **d** flower; **e**: berry

*Sambucus williamsii* is a shrub or small tree growing to a height of 5–6 m (Fig. 2a) that is widely distributed in northeastern China. The aging branches become reddish-brown and exhibit narrowly elliptic lenticels on their surface (Fig. 2b). The leaves are imparipinnate with 2- or 3-jugate leaflets, which are ovate–orbicular or narrowly elliptic at 5–15 × 1.2–7 cm, and irregularly serrate margins (Fig. 2c). The stems terminate in a cymose panicle of 5–11 × 4–14 cm in diameter, with numerous white or yellowish flowers (Fig. 2d). The fruit is a small glossy red berry of 3–5 mm in diameter (Fig. 2e). *Sambucus williamsii* flowers from April to May, and the seeds ripen from September to October. The plant is mostly located along mountain slopes, scrub, stream sides, and roadsides at altitudes of 540–1600 m, and has high environmental adaptability [1, 17].

### Medicinal properties

The stem of *S. williamsii* has been used in Chinese medicinal formulae, in combination with other herbs, to treat bone fractures [18, 19]. Medicinal effects include relieving swelling and pain [19–21], promoting blood circulation [20, 21], and acting as an anti-inflammatory effect [21]. The other parts of *S. williamsii* such as the stem bark, root bark, fruit oil, and leaves have been investigated with various biological screening models [2–6, 22–46]. The root bark of *S. williamsii* exerted fracture healing effects [31, 34] similar to those of the stem while the other parts exhibited different effects, such as antifungal [22, 28], anti-inflammatory [33], anticancer [38], and antiaging [37] activities.

An extract of the stem prevented reductions in bone mass and bone strength induced by estrogen deficiency in ovariectomized (OVX) rats and mice [25–27], increased proliferation and differentiation of UMR-106 cells [4, 5, 30, 46], and induced differentiation of pluripotent stem cells into neurons [47]. A stem extract of *S. williamsii* exerted beneficial effects on the microarchitecture of trabecular bone and inhibitory effects on urinary calcium excretion in OVX mice by upregulating the ratio of osteoprotegerin to receptor activator of nuclear factor-κB ligand expression in bone obtained from OVX mice [26]. The stem extract exerted free radical-scavenging properties [23], reversed damage to the function of INS-1E β cells induced by alloxan, and increased insulin excretion [24], while the stem bark extract showed antifungal activities by damaging the fungal plasma membrane [2, 3, 22, 48]. The root bark extract exerted healing effects on rabbit bone fractures [6, 31, 34], inhibitory effects on xylene-induced mouse ear edema and carrageenan-induced rat paw edema, and analgesic properties in rats and mice [33]. A mechanistic study showed that an ethanol extract of the root bark promoted MC3T3-E1 cell proliferation and differentiation through the bone morphogenetic protein 2/Smad/p38/c-Jun N-terminal kinase/runt-related transcription factor 2 signaling pathway [35]. The fruit oil exhibited immune-boosting [36], anticancer [38], and memory-improving [39] effects in mice, and antihyperlipidemic [37, 40] and antiaging [37] effects in rats. Furthermore, the leaves extract exhibited antibacterial [44] and anti-inflammatory [45] effects. The details of the bioactivities and chemical components in various parts of *S. williamsii* are listed in Additional file 1 [2–7, 20–34, 36–67].

### Chemical composition and potential health effects

To date, publications have described chemical research on many parts of *S. williamsii*, including the stem, root bark, leaves, and berries. The chemicals discovered in these components currently include 59 lignans [2–4, 6, 22, 27, 28, 46, 49, 68], 26 terpenoids represented by 16 iridoids, two sesquiterpenoids, and eight triterpenoids [4, 6, 29, 30, 49–58], 13 phenolic acids [4, 5, 56, 58], seven aliphatic compounds [4, 7, 30, 50, 57], 50 essential oils [59, 60], and 23 other compounds [4, 6, 45, 50–52, 56, 58]. Furthermore, several minerals [61], amino acids [61], and natural pigments [62] were identified in the fruit of *S. williamsii*.

### Lignans

#### Chemical

**Fig. 3:**
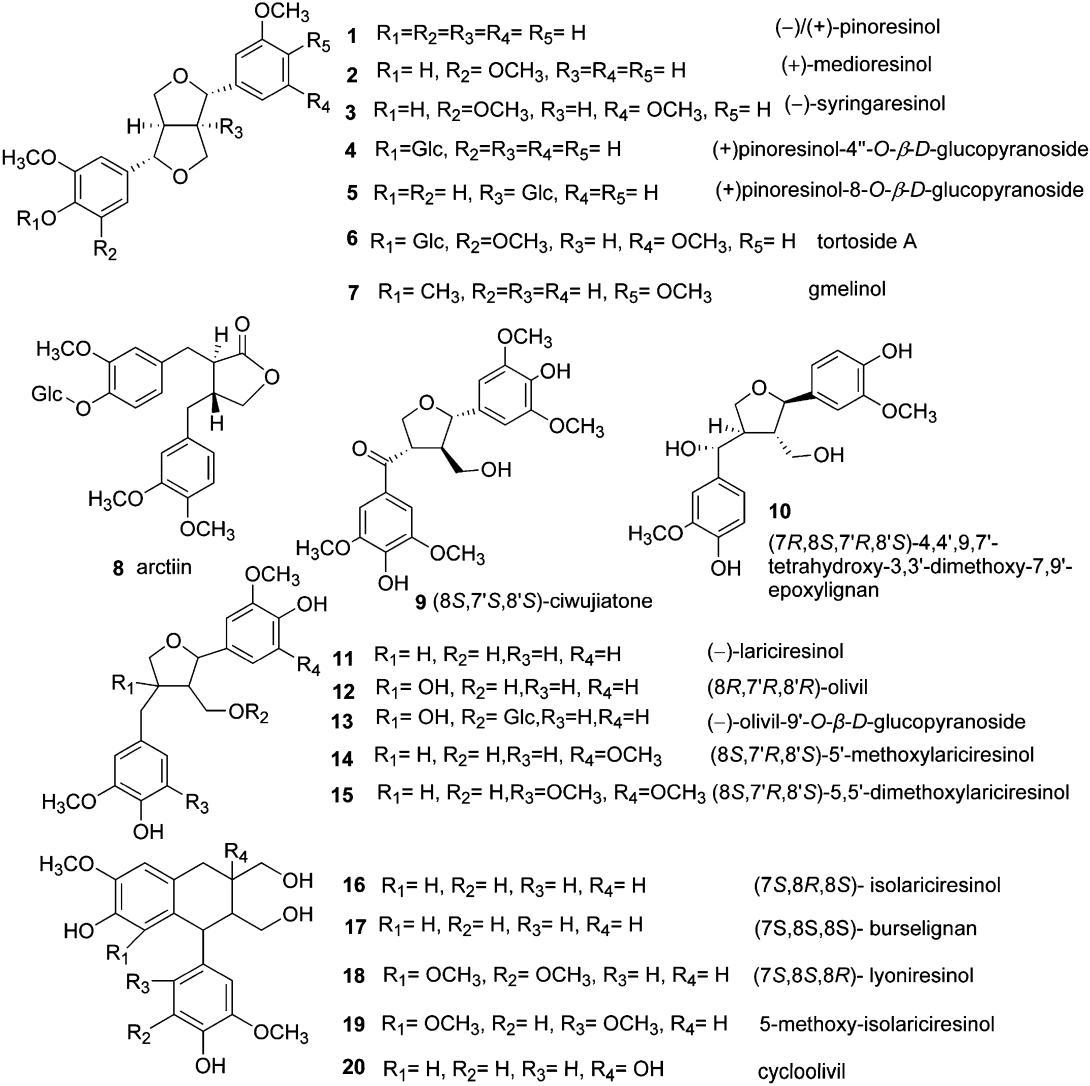
Chemical structures of lignans in *S. williamsii* with representative structures: classical types of lignans with an 8–8′ link

**Fig. 4:**
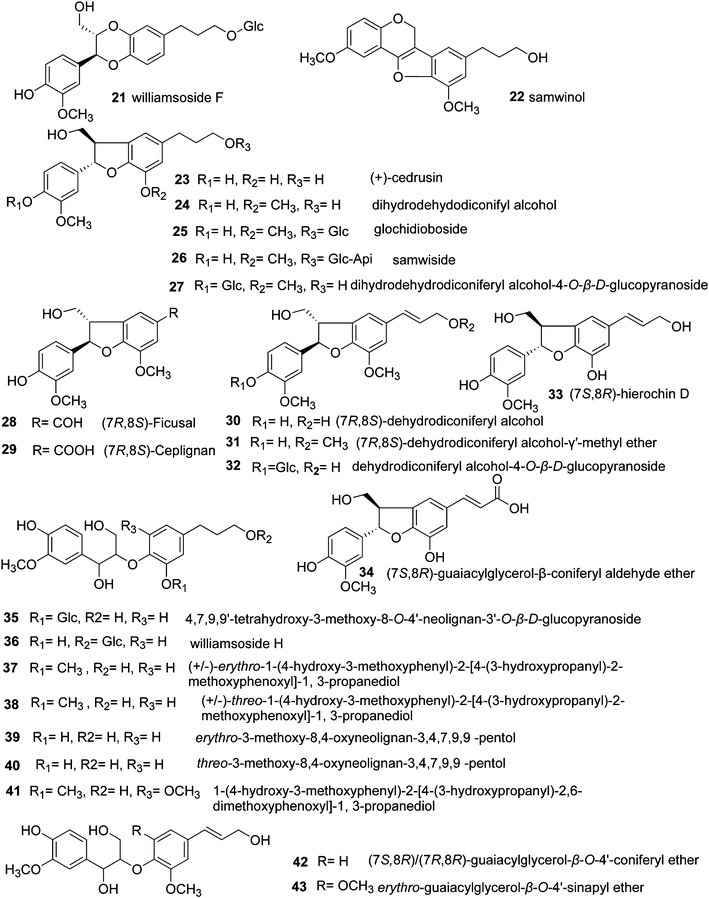
Chemical structures of lignans in *S. williamsii* with representative structures: neolignans with carbon linkages between C8–*O*–C3′/C7–*O*–C4′, C8–C3′/C7–*O*–C4′, and C8–*O*–C4′

**Fig. 5:**
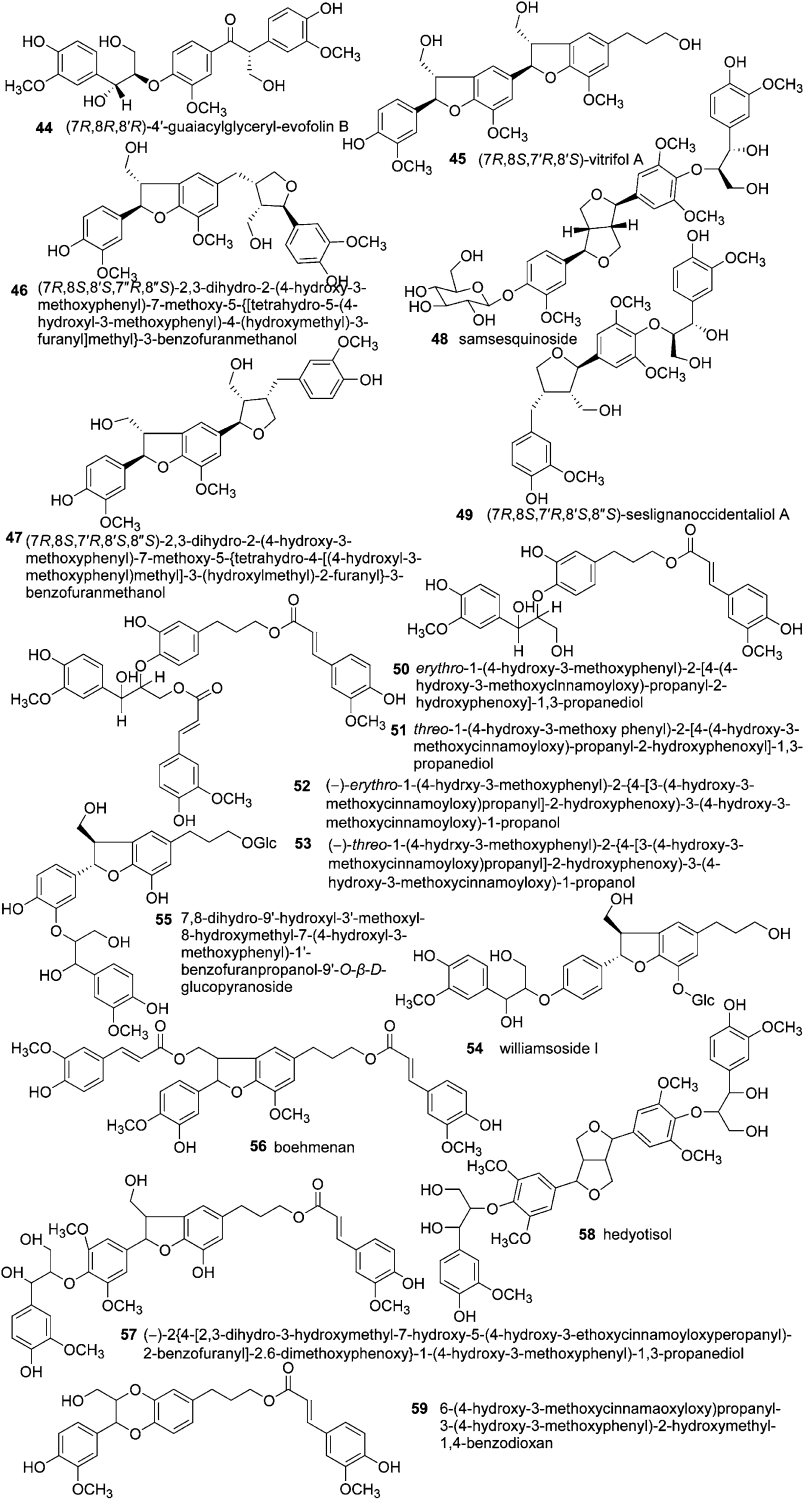
Chemical structures of lignans in *S. williamsii* with representative structures: oligomeric lignans

The lignans in *S.williamsii* include furofurans (**1**–**7**) [2, 4, 6, 28], dibenzyltyrolactones (**8**) [6], tetrahydrofurans (**9**–**15**) [4, 68], and arylnaphthalenes (**16**–**20**) [28, 63], representing the classical types of lignans, formed by oxidative coupling through a link between the β-carbons of the side chains of two phenylpropanoids (8–8′ link) [69] (Fig. 3). Benzodioxanes (**21**) [6], eupomatenoid benzofurans (**22**–**34**) [4, 6, 28, 46], and 8-*O*-4′ lignans (**35**–**43**) [6, 27, 28, 49, 63] are considered to be subtypes of neolignans, with carbon linkages between C8–*O*–C3′/C7–*O*–C4′, C8–C3′/C7–*O*–C4′, and C8–*O*–C4′, respectively (Fig. 4). Compounds **44**–**59** [4, 6, 28, 46] are oligomeric lignans composed of more than two C6–C3 units (Fig. 5). These lignans represent the most abundant compounds isolated from *S. williamsii*.

#### Health effects of lignans

These biphenolic compounds have similar structures to estrogens. They are the major source of phytoestrogens in the diets of Western populations and are primarily found in fiber-rich foods such as seeds, grains, vegetables, and fruits [70].

In the human gut, plant lignans are converted by intestinal bacteria into two enterolignans, enterolactone (ENL) and enterodiol (END), that exhibit biological activities and are absorbed into the bloodstream [71, 72]. Lignans also exhibit antiosteoporotic and antifungal effects [3] and can reduce the risk of cancer [73].

#### Osteoprotective effects

The potential therapeutic effects of *S. williamsii* on postmenopausal osteoporosis in animal models and their underlying mechanisms of action [25–27] have been investigated. The active compounds with potential osteoprotective effects were identified by biological assay-guided fractionation [27, 46, 74]. Specifically, an ethanol extract of the stem of *S. williamsii* exhibited protective effects on trabecular bone mass and mechanical strength of cortical bone in OVX rats fed a normal diet and mice fed a phytoestrogen-free AIN-93M diet [25, 26]. Moreover, the chemicals including lignans, phenolic acids and triterpenoids in the ethanol extract of *S. williamsii* stem stimulated osteogenesis by promoting osteoblastic proliferation and differentiation [25, 27, 46, 68].

A combination of 50 and 95 % aqueous ethanolic fractions from a crude extract of *S. williamsii* stem purified on a reverse-phase macroporous resin column was the mixture exhibiting the most potent antiosteoporotic activity [27]. Further isolation of the *S. williamsii* active fraction by a series of chromatography steps and preparative high-performance liquid chromatography led to the separation and identification of 55 lignans [27, 28, 46, 49, 63].

In vitro experiments [75] revealed that one of these lignans, compound **38**, exhibited estrogen-like effects in osteoblast-like UMR-106 cells, MC3T3-E1 cells, and bone mesenchymal stem cells. The results also showed that compound **38** exerted biological actions in osteoblast-like cells through ligand-independent, estrogen response element-independent, and mitogen-activated protein kinase-mediated rapid nongenomic estrogen receptor signaling pathways [75].

#### Antifungal activity

Pinoresinol (**1**), lariciresinol (**11**), (−)-olivil-9′-*O*-β-d-glucopyranoside (**13**), and glochidioboside (**25**) were all isolated from *S. williamsii*. They exhibited antifungal effects on human pathogenic strains through a membrane-disrupting action [2, 3, 22, 48]. (+)-Medioresinol (**2**), a furofuran-type lignan, isolated from the stem bark of *S. williamsii*, also exerted antifungal effects, but through the accumulation of reactive oxygen species in mitochondria [68].

#### Anticancer activity

Several studies [76, 77] showed that increased dietary lignan intake and/or increased levels of ENL and/or END might protect against or reduce the risk of breast, colon, and prostate cancers, and reduce hair loss. Lignans and their related metabolites were believed to be partly responsible for the growth inhibition of human prostate cancer cell lines [77]. ENL and END significantly inhibited the growth of prostate cancer PC-3 and LNCaP cells with 50 % inhibitive concentration at 57 and 100 μM respectively [77]. Treatment of human colon cancer SW480 cells with ENL and END, either alone or in combination, resulted in dose- and time-dependent decreases in cell number [78].

The administration of plant lignans, which were further metabolized to ENL and END, inhibited or delayed the onset of mammary cancer [71]. Although the mechanism of the anticarcinogenic action of ENL is not yet fully understood, there is intriguing evidence for ENL as a modulator of estrogen signaling [71]. Consumption of lignans such as lariciresinol (**11**) and pinoresinol (**1**) was associated with a significant reduction in breast cancer risk according to the clinical results of premenopausal women in Mexico [79].

### Phenolic acids

#### Chemical characteristics

**Fig. 6:**
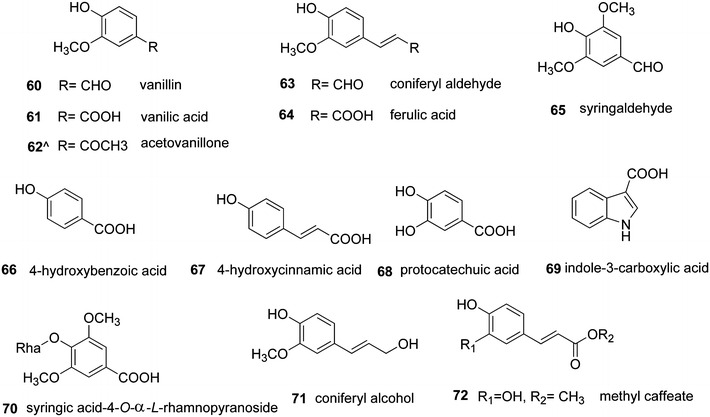
Chemical structures of phenolic acids present in *S. williamsii*

Thirteen phenolic compounds, vanillin (**60**), vanillic acid (**61**), acetovanillone (**62**), coniferyl aldehyde (**63**), ferulic acid (**64**), syringaldehyde (**65**), 4-hydroxybenzoic acid (**66**), 4-hydroxycinnamic acid (**67**), protocatechuic acid (**68**), indole-3-carboxylic acid (**69**), syringic acid-4-*O*-α-l-rhamnopyranoside (**70**), coniferyl alcohol (**71**), and methyl caffeate (**72**), were isolated from the stem and root bark of *S. williamsii* (Fig. 6) [5, 27, 28, 56, 58].

#### Health benefits of phenolic acids

Vanillic acid (**61**) exerts estrogen-like actions in osteoblastic-like cells through a nongenomic estrogen receptor signaling pathway involving the mitogen-activated protein kinase pathway [80]. The compound also exhibits antibacterial [81] and antimicrobial [82] activities and chemopreventive effects in experimentally induced carcinogenesis [83]. The protective effects of vanillic acid on myocardial infarction were studied in isoproterenol-induced cardiotoxic rats [84]. The free radical-scavenging, antioxidant, and anti-inflammatory activities of vanillic acid reduced isoproterenol-induced oxidative stress, downregulated myocardial interleukin-1β, interleukin-6, and tumor necrosis factor-α gene expression, and inhibited inflammation, thereby preventing cell death and protecting the myocardium [84].

Ferulic acid (**64**) possesses high antioxidant capacity and exhibits a longer residence time in rats than vitamin C [85]. Ferulic acid exhibits a wide range of therapeutic effects against many chronic conditions, including inflammation, cancer, apoptosis, diabetes, cardiovascular diseases, and neurodegenerative diseases [86]. It may also assist in plant host defense against pathogens and pests [87].

Protocatechuic acid (**68**) is an effective agent in reducing the carcinogenic actions of diethyl nitrosamine in the liver [88], 4-nitroquinoline-l-oxide in the oral cavity [89], azoxymethane in the colon [90], *N*-methyl-*N*-nitrosourea in the glandular stomach tissue [91], and *N*-butyl-*N*-(4-hydroxybutyl) nitrosamine in the bladder [92]. Protocatechuic acid also exhibits protective effects against the oxidative damage induced by tert-butyl hydroperoxide in rat primary hepatocytes by quenching free radicals [93]. Syringaldehyde (**65**) has six times higher antioxidant activity than protocatechuic acid [94]. Furthermore, syringaldehyde exerts antifungal activity against *Candida guilliermondii* [95], and exhibits antioncogenic activity through its inhibitory actions on murine pulmonary and hepatic microsomes [96]. Syringaldehyde shows stimulatory effects on both proliferation and alkaline phosphatase activity in UMR-106 cells [5]. 4-Hydroxybenzoic acid (**67**) exerts a hypoglycemic effect and increases serum insulin levels and liver glycogen contents in normal rats after oral administration at 5 mg/kg [97].

### Terpenoids

#### Chemical characteristics

**Fig. 7:**
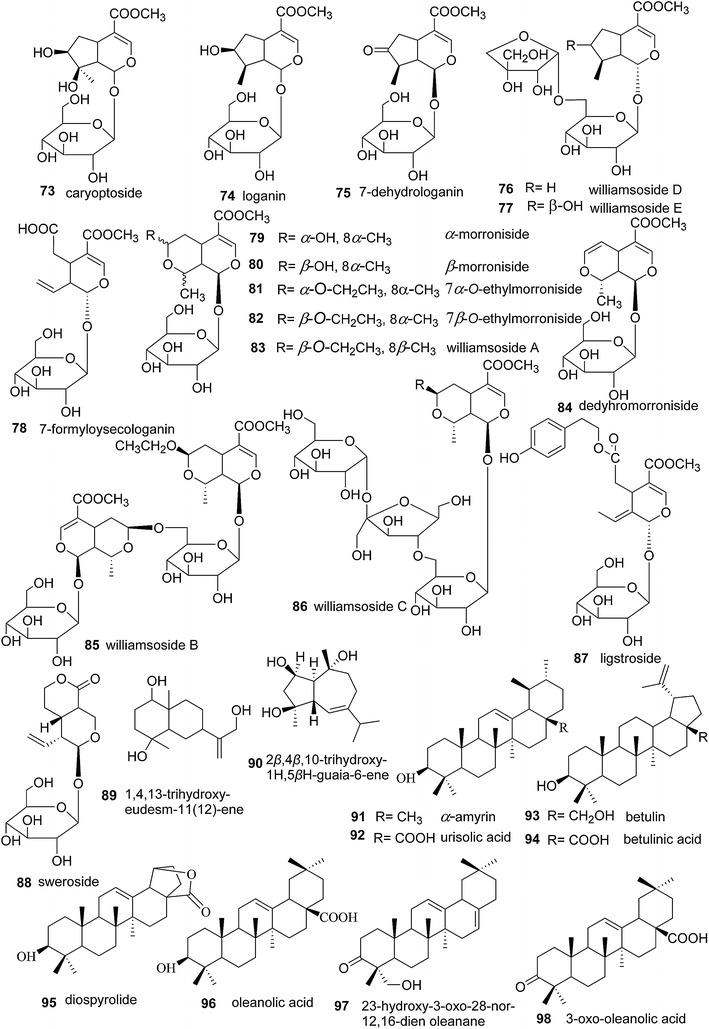
Chemical structures of terpenoids present in *S. williamsii*

Sixteen iridoids [6, 49, 51, 52, 54, 56], two sesquiterpenoids [4, 30, 58], and eight triterpenoids [4, 29, 50, 57] were identified in *S. williamsii* (Fig. 7). The iridoids are characterized by the presence of a partially hydrogenated *cis*-fused cyclopenta [c] pyran system, arising from intramolecular acetylation of a 1,5-cyclopenta dialdehyde moiety, and they are usually stabilized by acetylation or esterification. Iridoids can be subdivided into four groups: iridoid glycosides, simple iridoids or non-glycosidic iridoids, secoiridoids, and bisiridoids [98]. Compounds **73**–**77** were isolated as iridoid glycosides possessing a 9-carbon skeleton with glycosides linked to C1–OH. Compounds **78**–**88** belong to the secoiridoid subclass indicated by a bond-break between C7 and C8. Compounds **89** and **90** are the two sesquiterpenoids that have been isolated from *S. williamsii*. The eight triterpenoids are compounds **91**–**98** and represent three subclasses: urane (**91**, **92**), lupine (**93**–**95**), and oleanane (**96**–**98**).

#### Health benefits of terpenoids

Triterpenoids from plants possess a wide spectrum of pharmacological activities such as anti-inflammatory, antiulcer, antihyperlipidemic, antitumor, and hepatoprotective actions [99, 100]. α-Amyrin (**91**) possesses antimicrobial, anti-inflammatory, gastroprotective, and antinociceptive properties [8, 9], while betulinic acid (**94**) exhibits anti-inflammatory [101], antiviral [10], antidiabetic [11], antimalarial [12], and antitumor [10] activities.

### Aliphatic compounds

#### Chemical characteristics

**Fig. 8:**
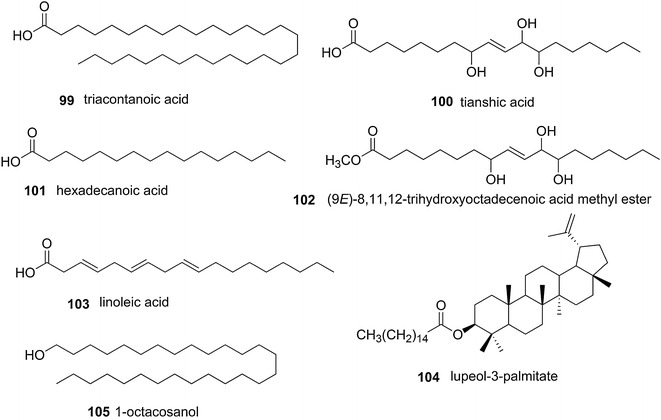
Chemical structures of aliphatic compounds present in *S. williamsii*

Seven aliphatic compounds, triacontanoic acid (**99**), tianshic acid (**100**), hexadecanoic acid (**101**), (9*E*)-8,11,12-trihydroxyoctadecenoic acid methyl ester (**102**), linoleic acid (**103**), lupeol-3-palmitate (**104**), and 1-octacosanol (**105**), were isolated and identified from the stem of *S. williamsii* (Fig. 8) [4, 7, 30, 50, 57].

#### Health benefits of aliphatic compounds

Linoleic acid (**103**) extracted from *S. williamsii* seed oil with a yield of 65.81 % possesses antioxidant, antiglycemic, and hypolipidemic activities [7]. It exerts free radical-scavenging activity at 61.9 mg/mL, inhibits the activity of α-glucosidase at 1.5–25 mg/mL, and significantly improves serum lipid levels in hyperlipidemic mice [7].

Lupeol-3-palmitate (**104**) significantly reduced prostaglandin E2 production in A23187-stimulated macrophages [102]. The anti-inflammatory effect of a lupeol-rich extract was similar to that exhibited by the selective cyclooxygenase inhibitor indomethacin [102, 103].

Saturated aliphatic compounds are known to have harmful effects on human health, but only trace amounts of saturated aliphatic compounds have been identified in *S. williamsii*. Hexadecanoic acid (**101**) found in *S. williamsii* induces oxidative stress and apoptosis of insulin-secreting cells [104, 105] and causes cardiac cells to undergo apoptosis [106]. It also causes insulin resistance in the brain by impairing the ability of insulin to activate intracellular signaling pathways [107], and accelerates obesity with diets containing high amounts of hexadecanoic acid [107].

### Other compounds

Fifty essential oils in *S. williamsii* were extracted by steam distillation and identified by gas chromatography-mass spectrometry, as listed in Table 1. Among them, *cis*-3-hexenyl-3-methylbutanoate and salicylic acid methyl ester were the major components [60].

**Table 1:** The structures and molecular formula of essential oils in *S. williamsii*

No.	Compound	Molecular formula	Relative amount (%)
1	Hexanal	C_6_H_12_O	0.05
2	α-Terpineol	C_10_H_18_O	0.06
3	4-Terpineol	C_10_H_18_O	0.08
4	α-Pinene	C_10_H_16_	0.09
5	Camphor	C_10_H_16_O	0.11
6	δ-Elemene	C_15_H_24_	0.11
7	Heneicosane	C_21_H_44_	0.21
8	2-Pentadecanone	C_15_H_30_O	0.24
9	3-Methyl-pentanoic acid methyl ester	C_7_H_14_O_2_	0.26
10	6,10-Dimethyl-5,9-undecadien-2-one	C_13_H_22_O3	0.38
11	Diallyl disulphide	C_6_H_12_S_2_	0.41
12	Eicosane	C_20_H_42_	0.42
13	3-Vinyl-1,2-dithio cyclohe-5-ene	C_7_H_10_S_2_	0.44
14	Thymol	C_10_H_14_O	0.48
15	β-Ionone	C_13_H_20_O	0.48
16	Hexadecane	C_16_H_34_	0.64
17	Epi-bicyclosequiphellandrene	C_15_H_24_	0.69
18	Ethyl salicylate	C_9_H_10_O_3_	0.69
19	2,4,10,14-Tetramethyl pentadecane	C_20_H_42_	0.76
20	Hexanoic acid 2-hexenyl ester	C_12_H_22_O_2_	0.83
21	Heptadecane	C_17_H_36_	0.85
22	Hyacinthin	C_8_H_8_O	0.87
23	Dihydro-β-agarofuran	C_15_H_26_O	0.89
24	3-Methyl-pentanoic acid	C_6_H_12_O_2_	1.10
25	1,2-Dithiolane,1,1-dioxide	C_3_H_6_O_2_S_2_	1.26
26	Decanal	C_8_H_16_O	1.30
27	1-Heptan-3-ol	C_7_H_14_O	1.33
28	Ethyl caproate	C_8_H_16_O_2_	1.34
29	Isoamyl isovalerate	C_10_H_20_O_2_	1.52
30	Heptanal	C_7_H_14_O	1.53
31	Benzaldehyde	C_7_H_6_O	1.85
32	Cyclotetradecane	C_14_H_28_	1.95
33	Hexanoic acid hexyl ester	C_12_H_24_O_2_	1.96
34	l-Linalool	C_10_H_18_O	2.04
35	Isovaleric acid	C_5_H_10_O_2_	2.08
36	3-Methyl-1-butanol	C_5_H_12_O	2.11
37	Octanal	C_7_H_14_O	2.11
38	*cis*-3-Hexenol	C_6_H_12_O	2.19
39	trans-2-Hexenyl isovalerate	C_11_H_20_O_2_	2.23
40	Benzyl isovalerate	C_12_H_16_O_2_	3.04
41	4-Methoxy-6-(2-propenyl)-1,3-benzodioxole	C_18_H_18_O_3_	3.10
42	2-Phenylethyl-3-methylbutanoate	C_13_H_18_O_2_	3.11
43	*cis*-3-Hexenyl caproate	C_12_H_22_O_2_	3.24
44	3-Methyl-butanoic acid ethyl ester	C_7_H_14_O_2_	3.68
45	2-Heptanone	C_7_H_14_O	3.86
46	Hexyl isovalerate	C_11_H_22_O_2_	4.02
47	1-Methoxy-4-(1-propenyl)benzene	C_10_H_12_O	6.29
48	1-Methoxy-4-(2-propenyl)benzene	C_10_H_12_O	6.79
49	*cis*-3-Hexenyl-3-methylbutanoate	C_11_H_20_O_2_	14.03
50	Salicylic acid methyl ester	C_8_H_8_O_3_	22.89

**Fig. 9:**
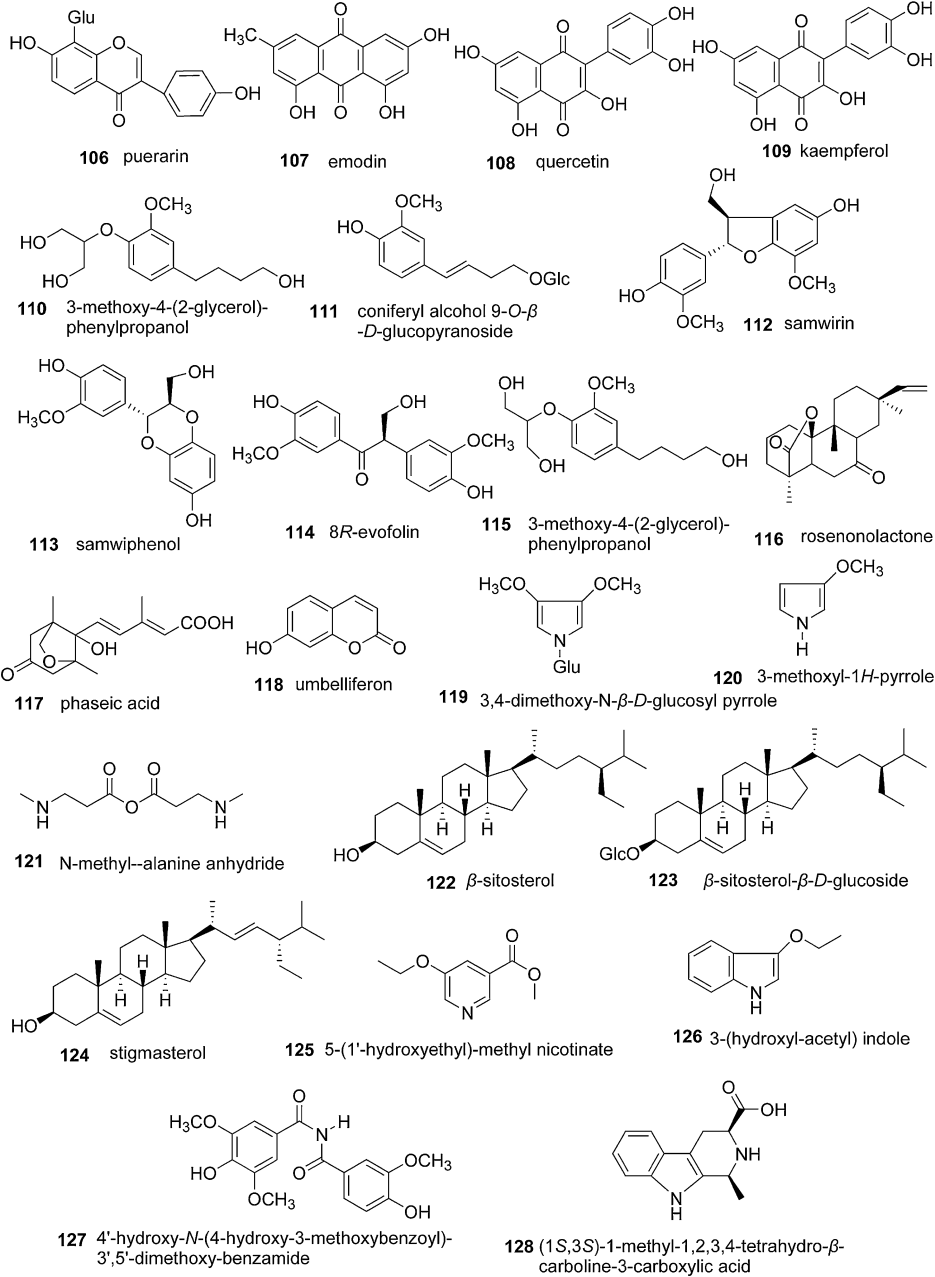
Chemical structures of other compounds also present in *S. williamsii*

Several isoflavonoids, anthraquinones, steroids, alcohols, ketones, phenylpropanoids, acids, coumarins, and nitrogen-containing compounds were isolated from the stem and root bark of *S. williamsii* (Fig. 9). These compounds included puerarin (**106**), emodin (**107**), quercetin (**108**), kaempferol (**109**), 3-methoxy-4-(2-glycerol)-phenylpropanol (**110**), coniferyl alcohol 9-*O*-β-d-glucopyranoside (**111**), samwirin (**112**), samwiphenol (**113**), 8*R*-evofolin (**114**), 3-methoxy-4-(2-glycerol)-phenylpropanol (**115**), rosenonolactone (**116**), phaseic acid (**117**), umbelliferone (**118**), 3,4-dimethoxy-*N*-β-d-glucosyl pyrrole (**119**), 3-methoxyl-1*H*-pyrrole (**120**), *N*-methyl-β-alanine anhydride (**121**), β-sitosterol (**122**), β-sitosterol-β-d-glucoside (**123**), stigmasterol (**124**), 5-(1′-hydroxyethyl)-methyl nicotinate (**125**), 3-(hydroxyl-acetyl)indole (**126**), 4′-hydroxy-*N*-(4-hydroxy-3-methoxybenzoyl)-3′,5′-dimethoxy-benzamide (**127**), and (1*S*,3*S*)-1-methyl-1,2,3,4-tetrahydro-β-carboline-3-carboxylic acid (**128**) [4, 6, 29, 45, 50, 51, 56, 58]. Du et al. [61] systemically studied the berries of *S. williamsii* and identified 17 amino acids and 14 microelements (Table 2), which may account for the fruit’s nutritional properties.

**Table 2:** The components of amino acids and microelements in *S. williamsii*

Amino acid	Content (mg/100 mL)
Aspartic acid	8.295
Threonine	2.253
Serine	3.145
Glutamic acid	9.759
Glycine	2.876
Alanine	3.152
Cysteine	0.420
Valine	2.615
Methionine	0.451
Isoleucine	2.158
Leucine	3.588
Tyrosine	1.784
Phenylalanine	2.216
Lysine	3.301
Histidine	1.279
Arginine	2.917
Proline	3.946

Microelements	Content (μg/g)
K	752
Ca	52.9
Zn	1.53
Fe	5.71
Cr	0.09
Cu	0.99
Mn	0.48
Ni	0.04
P	145
Sr	0.39
Ti	0.35
V	0.01
Al	10.4
Ba	0.46

## Conclusions

This article reviewed the phytochemicals identified from *S. williamsii*, together with their biological activities and potential health benefits. Although several biological activities were ascribed to *S. williamsii*, the most important beneficial effects identified to date, based on the biological evidence outlined in this review, are those in the areas of osteoporosis, bone fractures, and other bone-related diseases.

## Additional file

10.1186/s13020-016-0106-9 The bioactivities and components of different usage parts of *S. williamsii.*

## Abbreviations

END: enterodiol
ENL: enterolactone
OVX: ovariectomized
*S. williamsii*: *Sambucus williamsii* Hance

## References

[1] Song LR (2000). The Editorial Committee of Chinese Herbals State Administration of Traditional Chinese Medicine.

[2] Choi H, Lee J, Chang YS, Woo ER, Lee DG (2013). Isolation of (−)-olivil-9′-*O*-β-d-glucopyranoside from *Sambucus williamsii* and its antifungal effects with membrane-disruptive action. Biochim Biophys Acta.

[3] Hwang B, Cho J, Hwang IS, Jin HG, Woo ER, Lee DG (2011). Antifungal activity of lariciresinol derived from *Sambucus williamsii* and their membrane-active mechanisms in Candida albicans. Biochem Biophys Res Commun.

[4] Yang XJ. The study of antiosteoporosis constituents of *Sambucus williamsii* Hance. Doctoral thesis. Shenyang Pharmaceutical University. 2005. p. 97–100.

[5] Yang XJ, Wong MS, Wang NL, Chan SC, Yao XS (2005). Effect of phenolic acids isolated from *Sambucus williamsii* on proliferation and differentiation of rat osteoblastic UMR106 cells. Chin Tradit Herb Drugs.

[6] Han H. Studies on chemical constituents and pharmacological effect of active fraction for fracture healing in *Sambucus*. Doctoral thesis. Heilongjiang University of Chinese Medicine. 2006.

[7] Lv H, Chen SS, Xu XL, Zhu MM, Zhao WF, Liu KW, Liu KH (2015). Isolation of linoleic acid from *Sambucus williamsii* seed oil extracted by high pressure fluid and its antioxidant, antiglycemic, hypolipidemic activities. Int J Food Eng.

[8] Oliveira FA, Chaves MH, Almeida FRC, Lima RCP, Silva RM, Maia JL, Brito GA, Santos FA, Rao VS (2005). Protective effect of α-and β-amyrin, a triterpene mixture from *Protium heptaphyllum* (Aubl.) March. trunk wood resin, against acetaminophen-induced liver injury in mice. J Ethnopharmacol.

[9] Lima-Júnior RCP, Sousa DIM, Brito GA, Cunha GM, Chaves MH, Rao VS, Santos FA (2007). Modulation of acute visceral nociception and bladder inflammation by plant triterpene, α, β-amyrin in a mouse model of cystitis: role of tachykinin NK1-receptors, and K(+)ATP channels. Inflamm Res.

[10] Pisha E, Chai H, Lee IS, Chagwedera TE, Farnsworth NR, Cordell GA, Beecher CWW, Fong HHS, Kinghorn AS, Browh DM, Wani MC, Wall ME, Hieken TJ, Gupta TKD, Pezzuto JM (1995). Discovery of betulinic acid as a selective inhibitor of human melanoma that functions by induction of apoptosis. Nat Med.

[11] De Melo CL, Queiroz MG, Arruda Filho ACV, Rodrigues AM, De Sousa DF, Almeida JG, Pessoa OD, Silveira ER, Menezes DB, Melo TS, Santos FA, Rao VS (2009). Betulinic acid, a natural pentacyclic triterpenoid, prevents abdominal fat accumulation in mice fed a high-fat diet. J Agric Food Chem.

[12] Bringmann G, Saeb W, Assi LA, Francois G, Sankara NAS, Peters K, Peters KM (1997). Betulinic acid: isolation from *Triphyophyllum peltatum* and *Ancistrocladus heyneanus*, antimalarial activity, and crystal structure of the benzyl ester. Planta Med.

[13] Eriksson T, Dongohue MJ (1997). Phylogenetic relationships of *Sambucus* and Adoxa (Adoxoideae, Adoxaceae) based on nuclear ribosomal ITS sequences and preliminary morphological data. Syst Bot.

[14] von Schwerin FG. Mitteilungen der Deutschen Dendrologischen Gesellschaft. In: Monographie der gattung *Sambucus*. Nabu Press. 1909. p. 1–56.

[15] von Schwerin FG. Mitteilungen der Deutschen Dendrologischen Gesellschaft. In: Revisio generis Sambucus. Nabu Press. 1920. p. 57-94.

[16] Bolli R (1994). Revision of the genus *Sambucus*. Diss Bot.

[17] Editoral Committee of Flora Repubulicae Popularis Sinicae (1988). Flora Repubulicae Popularis Sinicae.

[18] Lin M, Mei J, Liu X (2009). The clinical observation of the effects of traditional chinese medicine on healing fracture. J Mod Med Health.

[19] Lin M, Mei J (2010). The clinical observation of compound JGM capsule on healing femur neck fracture. Zhongguo Zhongyi Jizheng.

[20] Song GH, Jiang B (2009). Investigation of the major pharmacodynamic effects of compound JGM capsule. Chongqing Zhong Cao Yao Yanjiu.

[21] Liu XW, Mei J, Lin M, Yang XD, Jiang B (2010). Investigation of the major pharmacological effects of compound JGM capsules. Xin Zhong Yi.

[22] Hwang B, Lee J, Liu QH, Woo ER, Lee DG (2010). Antifungal effect of (+)-pinoresinol isolated from *Sambucus williamsii*. Molecules.

[23] Li AL, Xiong SL (2010). Extraction technology and free radical scavenging properties of flavanone from *Sambucus williamsii* hance. Zhongguo Shipin Tian Jia Ji.

[24] Song LL, Fu JN (2011). Effects of *Sambucus* polysaccharides on rat cell proliferation and insulin secretion. Zhongguo Yao Li Xue Tong Bao.

[25] Xie F, Wu CF, Zhang Y, Yao XS, Cheung PY, Chan AS, Wong MS (2005). Increase in bone mass and bone strength by *Sambucus williamsii* HANCE in ovariectomized rats. Biol Pharm Bull.

[26] Zhang Y, Li Q, Wan HY, Xiao HH, Lai WP, Yao XS, Wong MS (2011). Study of the mechanisms by which *Sambucus williamsii* HANCE extract exert protective effects against ovariectomy-induced osteoporosis in vivo. Osteoporos Int.

[27] Xiao HH, Dai Y, Wan HY, Wong MS, Yao XS (2011). Bone-protective effects of bioactive fractions and ingredients in *Sambucus williamsii* HANCE. Br J Nutr.

[28] Xiao HH. Chemical constituents with antiosteoporosis effects in the bioactive fraction of *Sambucus willamsii* Hance. Doctoral thesis. Shenyang Pharmaceutical University. 2011.

[29] Yang XJ, Wang NL, Wong MS, Chan SC, Yao XS (2005). Studies of triterpenoids isolated from *Sambucus williamsii* Hance and their effects on UMR106 cell proliferation and alkaline phosphatase activity. Shenyang Yao Ke Da Xue Xuebao.

[30] Yang XJ, Wong MS, Wang NL, Chan SC, Yao XS (2006). A new eudesmane derivative and a new fatty acid ester from *Sambucus williamsii*. Chem Pharm Bull.

[31] Han H, Yang BY, Xia YG, Wang QH, Kuang HX (2013). Pharmacological Mechanism of *Sambucus williamsii* Hance in promoting fracture healing. Zhongguo Yao Shi.

[32] Han H, Yang BY, Yang L, Xia YG, Wang QH, Kuang HX (2013). Promotion of *Sambucus williamsii* root barks on fracture healing. Zhong Cao Yao.

[33] Dong PL, Yan XY, Kuang HX, Han H (2008). Experimental study of *Sambucus williamsii* Hance on anti-inflammatory and analgesia. Zhong Yi Yao Xuebao.

[34] Yang BY, He YW, Zhu XQ, Han H, Yang L, Wang QH (2014). Study on effects of *Sambucus williamsii* total glycosides tablets on fracture healing and inflammation: part I. Zhongguo Yao Shi..

[35] Yang BY, Lin XY, Yang CL, Tan JY, Li W, Kuang HX (2015). *Sambucus williamsii* Hance promotes MC3T3-E1 cells proliferation and differentiation via BMP-2/Smad/p38/JNK/Runx2 signaling pathway. Phytother Res..

[36] Wang QY, Li TM (1995). The study of fruit oil of *Sambucus williamsii* Hance on lymphocyte transformtion of mouse in vivo. Liaoning Da Xue Xuebao.

[37] Liu Z, Wu JS, Wang MW (1995). Effects of *Sambucus williamsii* Hance fruit oil on reducing plasma lipids and anti-aging. Shenyang Yao Ke Da Xue Xuebao.

[38] Li XW, Shen GZ, Zhang SY, Hu R, Chen ZA, Jin ZN (2000). Study of the anti-cancer effects of *Sambucus williamsii* Hance fruit oil. Zhongguo Zhong Yi Yao Ke Ji.

[39] Shen GZ, Hu R, Zhang SY, Chen ZA, Jin ZN (2000). The effects of *Sambucus williamsii* Hance fruit oil on the memory of mice. Zhongguo Zhong Yi Yao Ke Ji.

[40] Hu R, Hong HC, Ma DB, Zheng HY (2000). Effect of *Sambucus williamsii* Hance fruit oil on reduce plasma lipids. Beihua Da Xue Xuebao (Nat Sci).

[41] Hu R, Qi JZ, Xue ZP, Jiang BW (2005). A new medicinal and edible oil from a woody plant-*Sambucus williamsii*. Lin Ye Ke Xue.

[42] Hua RZ (1998). Five common therapy herbs forarthralgia. Zhongguo Min Jian Liao Fa.

[43] Zhang HL, Han CX, Yang XJ, Wang MC, Yang QE, Bu SH (2004). Study on chemical constituents and rat-killing activity of *Sambucus williamsii*. Xibei Zhi Wu Xuebao.

[44] Zhang T, Sun H, Wang GQ (2011). The antibacterial effects of *Sambucus williamsii* Hance extracts to *Botrytis cinerea* Per. Ex Fr. in vitro. Liao Cheng Da Xue Xuebao..

[45] Yang HM, Zheng YJ, Dai Y (2012). Investigation of the bioactive fraction and components of *Sambucus williamsii* Hance on anti-inflammatory. Shizhen Guo Yi Guo Yao.

[46] Xiao HH, Dai Y, Wong MS, Yao XS (2014). New lignans from the bioactive fraction of *Sambucus williamsii* Hance and proliferation activities on osteoblastic-like UMR106 cells. Fitoterapia.

[47] Liu SP, Hsu CY, Fu RH, Huang YC, Chen SY, Lin SZ (2015). *Sambucus williamsii* induced embryonic stem cells differentiated into neurons. Biomedicine.

[48] Lee H, Choi H, Ko HJ, Woo ER, Lee DG (2014). Antifungal effect and mode of action of glochidioboside against *Candida albicans* membranes. Biochem Biophys Res Commun.

[49] Ouyang F, Liu Y, Li R, Li L, Wang NL, Yao XS (2011). Five lignans and an iridoid from *Sambucus williamsii*. Chin J Nat Med.

[50] Guo XM, Zhang L, Quan JC, Hong YF, Liu MZ (1998). Studies on the chemical constituents of *Williams elder* (*Sambucus williamsii*). Chin Tradit Herbal Drugs.

[51] Lv F. Studies on the constituents of *Sambucus williamsii* Hance. Master thesis. Heilongjiang University of Chinese Medicine. 2002.

[52] Han MH. Studies on the constituents of *Sambucus williamsii* Hance and on the preparation process of its compound prescription. Master Thesis. Heilongjiang University of Chinese Medicine. 2003.

[53] Wang ZY, Han H, Yang BY, Xia YG, Kuang HX (2011). Two new iridoid glycosides from the root barks of *Sambucus williamsii* Hance. Molecules.

[54] Kuang HX, Han H, Yang BY, Yang L, Jiang H, Wang QH (2012). Two new iridoid glycosides from the root barks of *Sambucus williamsii* Hance. Molecules.

[55] Song DD, Yang BY, Yang L, Han H, Liu Y, Wang QH (2014). Chemical constituents from roots of *Sambucus williamsii* Hance. Zhong Yi Yao Xin Xi.

[56] Yang BY, Song DD, Han H, Yang L, Liu Y, Wang QH (2014). Chemical constituents from roots of *Sambucus williamsii* (I). Zhong Cao Yao.

[57] Xu MM, Duan YH, Dai Y, Wang ZZ, Xiao W, Yao XS (2013). A new nortriterpenoid from *Sambucus williamsii*. Zhong Cao Yao.

[58] Xiao HH, Dai Y, Wong MS, Yao XS (2015). Two new phenylpropanoids and one new sesquiterpenoid from the bioactive fraction of *Sambucus williamsii*. J Asian Nat Prod Res.

[59] Fu K, Fu GY, Luan FW, Bao YM, Zhang L (2008). Study on the component of essential oil from mongolian medicine *Sambucus williamsii* Hance by GC–MS. J Inner Mong Univ Natl (Nat Sci).

[60] Zhao Y, Jin J, Mao HB (2013). Analysis of volatile oil in *Sambucus williamsii* by SPME. Guangzhou Hua Gong.

[61] Du FG, Sun GR, Liu JH (1996). Analysis on nutrient composition of *Sambucus williamsii* Hance fruits. Zi Yuan Ke Xue.

[62] Hu R (1992). Study on pigment extraction of *Sambucus willamsii* Hance. Econ Forest Res.

[63] Ouyang F, Liu Y, Xiao HH, Yu HY, Wang NL, Yao XS (2009). Lignans from stems of *Sambucus williamsii*. Zhongguo Zhong Yao Za Zhi.

[64] Chen KG, Hu R, Jiang BW (1994). The preliminary experiment of natural pigments extraction from the peel of *Sambucus williamsii* Hance. Shipin Gong Ye Ke Ji.

[65] Lou GY, Zhao Q, Chi SJ, Qin GQ (1998). Study of the *Sambucus williamsii* Hance seed oil—a new rich source of a-linolenic acid. Zhongguo You Zhi.

[66] Li F (2007). Analysis on nutritional components from wild *Sambucus williamsii* Hance. Yunnan Hua Gong.

[67] Zhao MJ (2010). The clinical experience of using *Sambucus williamsii* Hance on osteoarthritis. Zhongguo Min Zu Min Jian Yi Yao.

[68] Hwang JH, Hwang I, Liu QH, Woo ER, Lee DG (2012). (+)-Medioresinol leads to intracellular ROS accumulation and mitochondria-mediated apoptotic cell death in *Candida albicans*. Biochimie.

[69] Ayres DC, Loike JD (1990). Lignans: chemical, biological and clinical properties.

[70] Buck K, Zaineddin AK, Vrieling A, Heinz J, Linseisen J, Flesch-Janys D (2011). Estimated enterolignans, lignan-rich foods, and fibre in relation to survival after postmenopausal breast cancer. Br J Cancer.

[71] Saarinen NM, Wärri A, Airio M, Smeds A, Mäkelä S (2007). Role of dietary lignans in the reduction of breast cancer risk. Mol Nutr Food Res.

[72] Adlercreutz H (2007). Lignans and human health. Crit Rev Clin Lab Sci.

[73] Milder IEJ, Kuijsten A, Arts ICW, Feskens EJM, Kampman E, Hollman PCH (2007). Relation between plasma enterodiol and enterolactone and dietary intake of lignans in a Dutch endoscopy-based population. J Nutr.

[74] Yang XJ, Wong MS, Wang NL, Chan SC, Yao XS (2007). Lignans from the stems of *Sambucus williamsii* and their effects on osteoblastic UMR106 cells. J Asian Nat Prod Res.

[75] Xiao HH, Gao QG, Ho MX, Zhang Y, Wong KC, Dai Y (2015). An 8-*O*-4′ norlignan exerts oestrogen-like actions in osteoblastic cells via rapid nongenomic ER signaling pathway. J Ethnopharmacol.

[76] Kuijsten A, Arts ICW, Vree TB, Hollman PCH (2005). Pharmacokinetics of enterolignans in healthy men and women consuming a single dose of secoisolariciresinol diglucoside. J Nutr.

[77] Lin X, Switzer BR, Demark-Wahnefried W (2000). Effect of mammalian lignans on the growth of prostate cancer cell lines. Anticancer Res.

[78] Qu H, Madl RL, Takemoto DJ, Baybutt RC, Wang W (2005). Lignans are involved in the antitumor activity of wheat bran in colon cancer SW480 cells. J Nutr.

[79] Torres-Sanchez L, Galvan-Portillo M, Wolff MS, Lopez-Carrillo L (2009). Dietary consumption of phytochemicals and breast cancer risk in Mexican women. Public Health Nutr.

[80] Xiao HH, Gao QG, Zhang Y, Wong KC, Dai Y, Yao XS (2014). Vanillic acid exerts oestrogen-like activities in osteoblast-like UMR 106 cells through MAP kinase (MEK/ERK)-mediated ER signaling pathway. J Steroid Biochem Mol Biol.

[81] Rai RP, Maurya MS (1996). Synthesis and evaluation of antibacterial activity of vanillin derivatives. J Sci Technol India.

[82] Delaquis P, Stanich K, Toivonen P (2005). Effect of pH on the inhibition of *Listeria* spp. by vanillin and vanillic acid. J Food Prot.

[83] Tsuda H, Uehara N, Iwahori Y, Asamoto M, Ligo M, Nagao M (1994). Chemopreventive effects of β-carotene, *α*-tocopherol and five naturally occurring antioxidants on initiation of hepatocarcinogenesis by 2-amino-3-methylimidazo [4,5-f] qumoline in the rat. Cancer Sci.

[84] Prince PSM, Rajakumar S, Dhanasekar K (2011). Protective effects of vanillic acid on electrocardiogram, lipid peroxidation, antioxidants, proinflammatory markers and histopathology in isoproterenol induced cardiotoxic rats. Eur J Pharmacol.

[85] Adam A, Crespy V, Levrat-Verny MA, Leenhardt F, Leuillet M, Demigné C, Rémésy C (2002). The bioavailability of ferulic acid is governed primarily by the food matrix rather than its metabolism in intestine and liver in rats. J Nutr.

[86] Srinivasan M, Sudheer AR, Menon VP (2007). Ferulic acid: therapeutic potential through its antioxidant property. J Clin Biochem Nutr.

[87] Mckeehen JD, Busch RH, Fulcher RG (1999). Evaluation of wheat (*Triticum aestivum* L.) phenolic acids during grain development and their contribution to *Fusarium* resistance. J Agric Food Chem.

[88] Tanaka T, Kojima T, Kawamori T, Yoshimi N, Mori H (1993). Chemoprevention of diethylnitrosamine-induced hepatocarcinogenesis by a simple phenolic acid protocatechuic acid in rats. Cancer Res.

[89] Tanaka T, Kawamori T, Ohnishi M, Okamoto K, Mori H, Hara A (1994). Chemoprevention of 4-nitroquinoline-1-oxide-induced oral carcinogenesis by dietary protocatechuic acid during initiation and postinitiation phases. Cancer Res.

[90] Kawamori T, Tanaka T, Kojima T, Suzui M, Ohnishi M, Mori H (1994). Suppression of azoxymethane-induced rat colon aberrant crypt foci by dietary protocatechuic acid. Cancer Sci.

[91] Tanaka T, Kojima T, Kawamori T, Mori H (1995). Chemoprevention of digestive organs carcinogenesis by natural product protocatechuic acid. Cancer.

[92] Hirose Y, Tanaka T, Kawamori T, Olnishi M, Makita H, Mori H (1995). Chemoprevention of urinary bladder carcinogenesis by the natural phenolic compound protocatechuic acid in rats. Carcinogenesis.

[93] Tseng TH, Wang CJ, Kao ES (1996). *Hibiscus* protocatechuic acid protects against oxidative damage induced by tert-butylhydroperoxide in rat primary hepatocytes. Chem Biol Interact.

[94] Bountagkidou OG, Ordoudi SA, Tsimidou MZ (2010). Structure-antioxidant activity relationship study of natural hydroxybenzaldehydes using in vitro assays. Food Res Int.

[95] Gurpilhares DB, Pessoa A, Roberto IC (2006). Glucose-6-phosphate dehydrogenase and xylitol production by *Candida guilliermondii* FTI 20037 using statistical experimental design. Process Biochem.

[96] Morse MA, Kresty LA, Toburen AL (1995). Inhibition of metabolism of 4-(methylnitrosamino)-1-(3-pyridyl)-1-butanone by dietary benzaldehydes. Cancer Lett.

[97] Peungvicha P, Temsiririrkkul R, Prasain JK, Tezuka Y, Kadota S, Thirawarapan SS (1998). 4-Hydroxybenzoic acid: a hypoglycemic constituent of aqueous extract of *Pandanus odorus* root. J Ethnopharmacol.

[98] Bianco A (1999). Recent developments in iridoids chemistry. Pure Appl Chem.

[99] Liu J, Liu YP, Parkinson A, Klaassen CD (1995). Effect of oleanolic acid on hepatic toxicant-activating and detoxifying systems in mice. J Pharmacol Exp Ther.

[100] James LP, Mayeux PR, Hinson JA (2003). Acetaminophen-induced hepatotoxicity. Drug Metab Dispos.

[101] Mukherjee PK, Saha K, Das J, Pal M, Saha BP (1997). Studies on the anti-inflammatory activity of rhizomes of *Nelumbo nucifera*. Planta Med.

[102] Fernández MA, Heras B, Garcia MD, Sáenz MT, Villar A (2001). New insights into the mechanism of action of the anti-inflammatory triterpene lupeol. J Pharm Pharmacol.

[103] Fernández A, Álvarez A, GarcíA MD, Sáenz MT (2001). Anti-inflammatory effect of *Pimenta racemosa* var. ozua and isolation of the triterpene lupeol. II Farmaco.

[104] Carlsson C, Hakan Borg LA, Welsh N (1999). Sodium palmitate induces partial mitochondrial uncoupling and reactive oxygen species in rat pancreatic islets in vitro. Endocrinology.

[105] Maedler K, Spinas GA, Dyntar D, Moritz W, Kaiser N, Donath MY (2010). Distinct effects of saturated and monounsaturated fatty acids on β-cell turnover and function. Diabetes.

[106] Sparagna GC, Hickson-Bick DL (1999). Cardiac fatty acid metabolism and the induction of apoptosis. Am J Med Sci.

[107] Posey KA, Clegg DJ, Printz RL, Byun J, Morton GJ, Vivekanandan-Giri A (2009). Hypothalamic proinflammatory lipid accumulation, inflammation, and insulin resistance in rats fed a high-fat diet. Am J Physiol Endocrinol Metab.

