# Supplementary material for: Phytochemicals and potential health effects of Sambucus williamsii Hance (Jiegumu)
Source: Chin Med. 2016 Jul 28;11:36. doi: 10.1186/s13020-016-0106-9 (PMC4965893; doi:10.1186/s13020-016-0106-9)
Supplement: Supplementary file 1 — 10.1186/s13020-016-0106-9 The bioactivities and components of different usage parts of S. williamsii. [file 13020_2016_106_MOESM1_ESM.doc]

**Additional file 1**

**The bioactivities and components of different usage parts of *S. williamsii***

| **Used part** | **Year** | **Components** | **Bioactivity** | **Reference** |
| --- | --- | --- | --- | --- |
| **Stem bark** | 2010 | (+)-pinoresinol (**1**) | Caused damage to the fungal plasma membrane | [22] |
|  | 2011 | (+)-pinoresinol (**1**) | Antifungal properties without hemolytic effects on human erythrocytes | [3] |
|  | 2013 | (-)-olivil glucopyranodise(**13**) | Antifungal activity | [2] |
|  | 2014 | Glochidioboside (**25**) | Antifungal effect with low hemolysis via a membrane disruptive mechanism | [48] |
| **Stem** | 1998 | *α*-amyrin (**91**), ursolic acid (**92**), triacontanoic acid (**99**), Lupeol-3-palmitate (**104**), *β*-sitosterol (**122**), *β*-sitosterol-*β*-*D*-glucoside (**123**) | / | [50] |
|  | 2005 | / | 60% ethanol extract of SWH prevented the reduction of bone mass and bone strength induced by estrogen deficiency in rats without substantial effects on uterus | [25] |
|  | 2005 | (-)-pinoresinol (**1**), (-)-syringaresinol (**3**), Tortoside A (**6**), (-)-lariciresinol (**11**), (-)-olivil (**12**), dehydrodehydodiconifyl alcohol (**30**), (-)-*erythro*-1-(4-hydroxy-3-methoxyphenyl)-2-[4-(3-hydroxypropanyl)-2-methyphenoxy]-1,3-propanediol (**37**), (-)-*threo*-1-(4-hydroxy-3-methoxyphenyl)-2-[4-(3-hydroxypropanyl)-2-methyphenoxy]-1,3-propanediol (**38**), (+)-3-methoxy-8,4′-oxyneolignan-3′,4,7,9,9′-pentol (**39**), (-)-guaiacylglycerol-*β*-*O*-4'-coniferyl (**42**), *erythro*-1-(4-hydroxy-3-methoxypheyl)-2-[4-(4-hydroxy-3-methoxycinnamoyloxy]propanyl-2-hydroxyphenoxy)-1,3-propanediol (**50**), *threo*-1-(4-hydroxy-3-methoxypheyl)-2-[4-(4-hydroxy-3-methoxycinnamoyloxy]propanyl-2-hydroxyphenoxy)-1,3-propanediol (**51**), (-)-*erythro*-1-(4-hydrxy-3-methoxyphenyl)-2-{4-[3-(4-hydroxy-3-methoxycinnamoyloxy)propanyl]-2-hydroxyphenoxy)-3-(4-hydroxy-3-methoxycinnamoyloxy)-1-propanol (**52**), (-)-*threo*-1-(4-hydrxy-3-methoxyphenyl)-2-{4-3(4-hydroxy-3-methoxycinnamoyloxy)propanyl]-2-hydroxyphenoxy)-3-(4-hydroxy-3-methoxycinnamoyloxy)-1-propanol (**53**), boehmenan (**56**), (-)-2{4-[2,3-dihydro-3-hydroxymethyl-7-hydroxy-5-(4-hydroxy-3-methoxycinnamoyloxyperopanyl)-2-benzofuranyl]-2.6-dimethoxyphenoxy}-1-(4-hydroxy-3-methoxyphenyl)-1,3-propanediol (**57**), hedyotisol (**58**), 6-(4-hydroxy-3-methoxycinnamaoxyloxy)propanyl-3-(4-hydroxy-3-methoxyphenyl)-2-hydroxymethyl-1,4-benzodioxan (**59**), vanillin (**60**), vanillic acid (**61**), acetovanillone (**62**), coniferyl aldehyde (**63**), ferulic acid (**64**), syringaldehyde (**65**), 4-hydroxybenzoic acid (**66**), 4-hydroxycinnamic acid (**67**), protocatechuic acid (**68**), indole-3-carboxylic acid (**69**), 1,4,13-trihydroxy-eudesm-11(12)-ene (**89**), α-amyrin (**91**), Ursolic acid (**92**),Betulin (**93**), betulinic acid (**94**), diospyrolide (**95**), oleanolic acid (**96**), tianshic acid (**100**), hexadecanoic acid (**101**), (9E)-8,11,12-trihydroxyoctadecenoic acid methyl ester (**102**), puerarin (**106**), Emodin (**107**), resenonolactone (**116**), phaseic acid (**117**), N-methyl--alanine anhydride (**121**), *β*-sitosterol-*β*-*D*-glucoside (**123**), Stigmasterol (**124**) | Proliferation effects and ALP activities on UMR 106 cells | [4] |
|  | 2005 | Vanillin (**60**), acetovanillone (**62**), coniferyl aldehyde (**63**), syringaldehyde (**65**), 4-hydroxybenzoic acid (**66**), 4-hydroxycinnamic acid (**67**), protocatechuic acid (**68**), | Proliferation and differentiation in UMR 106 cells | [5] |
|  | 2005 | *α*-amyrin (**91**), ursolic acid (**92**), Betulin (**93**), betulinic acid (**94),** oleanolic acid (**96**), *β*-sitosterol-*β*-*D*-glucoside (**123**), stigmasterol (**124**), | Proliferation and differentiation in UMR 106 cells | [29] |
|  | 2006 | 1,4,13-trihydroxy-eudesm-11(12)-ene (**89**), tianshic acid (**100**), 9(E)-8,11,12-trihydroxyoctadecenoic acid methyl ester (**102**) | ALP activity in UMR 106 cells | [30] |
|  | 2008/2013 | Essential oil (details in Table 1) | / | [59,60] |
|  | 2009 | isolariciresinol (**16**), burselignan (**17**), Lyoniresinol (**18**), 5-methoxy-isolariciresinol (**19**), cycloolivil (**20**), 1-(4-hydroxy-3-methoxyphenyl)-2-[4-(3-hydroxypropanyl)-2,6-dimethoxyphenoxyl]-1, 3-propanediol (**41**), *erythro*-guaiacylglycerol-*β*-*O*-4′-sinapyl ether (**43**) | / | [63] |
|  | 2010 | Flavanone | The properties of free radical scavenging | [23] |
|  | 2011 | Polysaccharides | Increased the proliferation of rat INS-1E β-cells significantly, reversed the damage on the function of INS-1E β-cells induced by AXN, and increased the insulin excretion. | [24] |
|  | 2011 | (7S,8R)-cedrusin (**23**), Dihydrodehydrodiconiferyl alcohol-4-*O*-*β*-*D*-glucopyranoside (**27**), Dehydrodiconiferyl alcohol-4-*O*-*β*-*D*-glucopyranoside (**32**), *erythro*-1-(4-hydroxy-3-methoxyphenyl)-2-[2-hydro-xy-4-(3-hydroxypropyl)phenoxy]-1,3-propanediol (**37**), *threo*-1-(4-hydroxy-3-methoxyphenyl)-2-[2-hydroxy-4-(3-hydroxypropyl) phenoxy]-1, 3-propanediol (**38**), and morroniside (**77**,**78**) | / | [49] |
|  | 2011 | / | SWH extract improved bone properties by inhibiting the process of bone resportion and stimulating the process of bone formation in mice | [26] |
|  | 2011 | (+)-lariciresinol (**11**), dihydrodehydrodiconiferyl alcohol (**24**), dehydrodiconiferyl alcohol (**30**), (+)-erythro-1-(4-hydroxy-3-methoxyphenyl)-2-[4-(3-hydroxypropanyl)-2-methoxy-phenoxy]-1,3-propanediol (**37**), (+)-thero-1-(4-hydroxy-3-methoxyphenyl)-2-[4-(3-hydroxypropanyl)-2-hydro-phenoxy]-1,3-propanediol (**38**), (+)-thero-guaiacylglycerol-*β*-*O*-4'-conifery ether (**42**), vanillic acid (**61**) | Bioactive fraction of SWH prevented bone loss, improved trabecular bone microstructure and increased cortical bone strength in ovariectomized (OVX) mice | [27] |
|  | 2011 | (7*R*,8*S*,7′*R*,8′*S*)-medioresinol (**2**), (7*R*,8*S*,7′*R*,8′*S*)-syringaresinol (**3**), (7*R*,8*S*,7′*R*,8′*S*)-tortoside A (**6**), Gmelinol (**7**), (8*S*,7′*S*,8′*R*)-ciwujiatone (**9**), (7*R*,8*R*,7′*R*,8′*S*)-4,4′,9,7′-tetrahydroxy-3,3′-dimethoxy-7.9′-epoxylignan (**10**), (8*S*,7′*R*,8′*S*)-lariciresinol (**11**), (8*S*,7′*R*,8′*S*)-olivil (**12**), (8*S*,7′*R*,8′*S*)-5′-methoxylariciresinol (**14**), (8*S*,7′*R*,8′*S*)-5,5′-dimethoxylariciresinol (**15**), (7*S*,8*R*,8′*S*)-isolariciresinol (**16**), (7*S*,8*S*,8′*S*)-Burselignan (**17**), (7*S*,8*S*,8′*R*)-Lyoniresinol (**18**), 3,9-dimethoxy-11-hydroxypropyl-6H-benzo-furo-[3,2-c]-[1]benzopyran (**22**), (7*R*,8*S*)-dihydrodehydrodiconiferyl alcohol (**24**), Samwiside (**26**), (7*R*,8*S*)-Ficusal (**28**), (7*R*,8*S*)-Ceplignan (**29**), (7*R*,8*S*)-dehydrodiconiferyl alcohol (30), (7*R*,8*S*)-dehydrodiconiferyl alcohol-γ′-methyl ether (**31**), (7*S*,8*R*)-dihydrodehydrodiconiferyl alcohol-9′-*O*-*β*-glucosdie (**32**), (7*S*,8*R*)-hierochin D (**33**), (7*S*,8*R*)- guaiacylglycerol-*β*-coniferyl aldehyde ether (**34**), (7*R*,8*R*)-1-(4-hydroxy-3-methoxyphenyl)-2-[4-(3-hydroxylpropyl)-2-methoxyphenoxy]-1,3-propanediol (**37**), (7*R*,8*S*)-1-(4-hydroxy-3-methoxyphenyl)-2-[4-(3-hydroxypropyl)-2-methoxyphenoxy]-1,3-propanediol (**38**), (7*R*,8*R*)- 3-methoxy-8,4′-oxyneolignan-3′,4,7,9,9′-pentol (**39**), (7*S*,8*R*)-3-methoxy-8,4′-oxyneolignan-3′,4,7,9,9′-pentol (**40**), (1*S*,2*R*)-1-(4′-hydroxy-3′-methoxyphenyl)-2-[4′′-(3-hydroxypropyl)-2′′,6′′-dimethoxyphenoxy]propane-1,3-diol (**41**), (7*S*,8*R*)-guaiacylglycerol-*β*-*O*-4'-conifery ether (**42**), (7*R*,8*R*)-guaiacylglycerol-*β*-*O*-4'-conifery ether (**42**), (7*S*,8*R*)-guaiacylglycerol-*β*-*O*-4′-sinapyl ether (**43**), (7*R*,7′′*R*,8′′*R*)-4′-guaiacyl-glycerol-Evofolin B (**44**), (7*R*,8*S*,7′*R*,8′*S*)-Vitrifol A (**45**), (7*R*,8*S*,8′*S*,7′′*R*,8′′*S*)-2,3-dihydro-2-(4-hydroxy-3-methoxyphenyl)-7-methoxy-5-{[tetrahydro-5-(4-hydroxy-3-methoxyphenyl)-4-(hydroxymethyl)-3-furanyl]methyl}-3-Benzofuranmethanol (**46**), (7*R*,8*S*,7′*R*,8′*S*,8′′*S*)-2,3-dihydro-2-(4-hydroxy-3-methoxyphenyl)-7-methoxy-5-{tetrahydro-4-[(4-hydro-xyl-3-methoxyphenyl)methyl]-3-(hydroxylmethyl)-2-furanyl}-3-Benzofuranmethanol (**47**), Samsesquinoside (**48**), (7*R*,8*S*,7′*R*,8′*S*,8′′*S*)- Seslignanoccidentaliol A (**49**) | Bioactive fraction of SWH exerted bone protective effects on mice and UMR 106 cells | [28] |
|  | 2013 | betulinic acid (**94**), Diospyrolide (**95**), oleanolic acid (**96**), 23-hydroxy-3-oxo-28-nor-12,16-dien oleanane (**97**), 3-oxo-oleanolic acid (**98**), hexadecanoic acid (**101**), 1-octacosanol (**105**) | / | [57] |
|  | 2014 | Samwinol (**22**), Samwiside (**26**), (7*R*,8*S*)-Ficusal (**28**), (7*R*,8*S*)-ceplignan (**29**), (7*R*,8*S*)-dehydrodiconiferyl alcohol (**30**), (7*R*,8*S*)-dehydrodiconiferyl alcohol-γ'-methyl ether (**31**), (7*S*,8*R*)-hierochin D (**33**), (7*R*,8*R*,8'*R*)-4'-guaiacylgluceryl-Evofolin B (**42**), (7*R*,8*S*,7'*R*,8'*S*)-Vitrifol A (**45**), (7*R*,8*S*,8'*S*,7''*R*,8''*S*)-2,3-dihydro-2-(4-hydroxy-3-methoxyphenyl)-7-methoxy-5-{[tetraydro-5-(4-hydroxy-3-methoxylphenyl)-4-(hydroxymethyl)-3-furanyl]methyl}-3-benzofuranmethanol (**46**), (7*R*,8*S*,7'*R*,8'*S*,8''*S*)-2,3-dihydro-2-(4-hydroxy-3-methoxyphenyl)-7-methoxy-5-[tetraydro-4-(4-hydroxy-3-methoxylphenyl)methyl-3-(hydroxylmethyl)-2-furanyl]-3-benzofuranmethanol (**47**), Samsequinoside (**48**), (7*R*,8*S*,7'*R*,8'*S*,8''*S*)-Seslignanoccidentaliol A (**49**) | Proliferation effects in UMR 106 cells | [46] |
|  | 2015 | / | The ethanol extract of SWH could induce pluripotent cells differentiated into neurons. It decreased Oct2, Sox2 (stem cells markers) genes expression while increased Tuj1 and Nestin genes expression. | [47] |
|  | 2015 | vanillic acid (**61**), ferulic acid (**64**), methyl caffeate (**72**), coniferyl alcohol (**71**), 2*β*,4*β*,10*α*-trihydroxy-1*α*H,5*β*H-guaia-6-ene (**90**), Samwirin (**112**), Samwiphenol (**113**), 8*R*-evofolin B (**114**), umbelliferon (**118**) | Proliferation effects on UMR 106 cells | [58] |
| **Root bark** | 2002 | *α*-morroniside (**79**), *β*-morriniside (**80**), 3,4-dimethoxy-*N*-*β*-*D*-glucosyl pyrrole (**119**) | / | [51] |
|  | 2003 | caryoptoside (**73**), *α*-morroniside (**79**), *β*-sitosterol (**122**), *β*-sitosterol-*β*-*D*-glucoside (**123**) |  | [52] |
|  | 2006 | (+)-pinoresinol-4''-*O*-*β*-*D*-glucopyranoside (**4**), (+)-pinoresinol-8-*O*-*β*-*D*-glucopyranoside (**5**), arctiin (**8),** williamsoside F (**21**), (7*S*,8*R*)-dihydrodehydodiconifyl alcohol (**24**), samwiside (26), glochidioboside (**25**), 4,7,9,9'-tetrahydroxy-3-methoxy-8-*O*-4'-isolignan-3'-*O*-*β*-*D*-glucopyranoside (**35**), williamsoside H (**36**), williamsoside I (**54**), caryoptoside (**73**), 7-dehydrologanin (**75**), williamsoside D (**76**), williamsoside E (**77**), 7-formyloysecologanin (**78**), *β*-morriniside (**80**), 7*α*-*O*-ethylmorroniside (**81**), 7*β*-*O*-ethylmorroniside (**82**), williamsoside A (**83**), williamsoside B (**85**), williamsoside C (**86**), ligstroside (**87**), 3-methoxy-4-(2-glycerol)-phenylpropanol (**110**) | Fracture healing in rabbit of bioactive fraction | [6] |
|  | 2008 |  | Obviously inhibitory effects on xylene induced mouse ear edema and carrageenan induced rat paw edema. It also raise mice’s threshold of pain. | [33] |
|  | 2011 | Williamsoside A (**83**), williamsoside B (**85**) | / | [53] |
|  | 2012 | Williamsoside D (**76**), Williamsoside C (**86**) | / | [54] |
|  | 2013 |  | The promoting effects of SWH on fracture healing are related with enhancement of collagen synthesis and inorganic salt deposition to improve the quality of callus | [31] |
|  | 2013 |  | Promoting the synthesis and secretion of ALP and BGP of osteoblast, promoting the synthesis of ACP in osteoclast, increasing the quantity of osteoblasts, promoting the absorption of necrotic bone, and speeding up the maturation calcification of bone matrix | [32] |
|  | 2014 | dihydrodehydodiconifyl alcohol (**24**), caryoptoside (**73**), *α*-morroniside (**79**), *β*-morroniside (**80**), ligstroside (**87**), *β*-sitosterol (**122**) | / | [55] |
|  | 2014 | 7,8-dihydro-9'-hydroxyl-3'-methoxyl-8-hydroxymethyl-7-(4-hydroxyl-*threo*-4,7,9,9'-tetrahydroxy-3-methoxy-8-*O*-4'-neolignan-3'-*O*-*β*-*D*-glucopyranoside (**35**), *threo*-1-(4-hydroxy-3-methoxyphenyl)-2-[2-hydroxy-4-(3-hydroxypropyl)phenoxy]-1,3-propanediol (**38**), 3-methoxyphenyl)-1'-benzofuranpropanol-9'-*O*-*β*-*D*-glucopyranoside (**55**), syringic acid-4-*O*-α-*L*-rhamnopyranoside (**70**), loganin (**74**), 7-dehydrologanin (**75**), 7-formylosecologanin (**78**), 7*α*-*O*-ethylmorroniside (**81**), 7*β*-*O*-ethylmorroniside (**82**), dehydromorroniside (**84**), sweroside (**88**), coniferyl alcohol 9-*O*-*β*-D-glucopyranoside (**111**), 3-methoxy-4-(2-glycerol)-phenylpropanol (**115**), 3-methoxyl-1*H*-pyrrole (**120**), 5-(1'-hydroxyethyl)-methyl nicotinate (**125**), 3-hydroxyl-acetyl indole (**126**), 4'-hydroxy-*N*-(4-hydroxy-3-methoxybenzoyl)-3',5'-dimethoxy-benzamide (**127**), (1*S*,3*S*)-1-methyl-1,2,3,4-tetrahydro-*β*-carboline-3-carboxylic acid (**128**) | / | [56] |
|  | 2014 | Total glycosides | Increased BMD and BMC in model group, the fracture site of model rabbit disappeared nearly; increased and deepened periosteal reaction density; relieved mice ear swelling of model mice and foot swelling of model rats. | [34] |
|  | 2015 | Ethanol extract | The ethanol extract of root bark could promote MC3T3-E1 cell proliferation and differentiation via BMP-2/Smad/p38/JNK/Runx2 signaling pathway | [35] |
| **Fruit** | 1992/1994 | Pigment | / | [62,64] |
|  | 1996 | Amino acids and microelements (details in Table 2) | / | [61] |
| **Fruit oil** | 1995 | / | Activate the lymphocyte and increase the immune function in Kunming mice | [36] |
|  | 1995 | / | Hypolipidemic effect and anti-aging in SD rats. Decreased cholesterol level in blood and prolong the survival time of hypoxia mice | [37] |
|  | 1998 | *α*-linolenic acid, *γ*-linolenic acid, linoleic acid (**103**) | / | [65] |
|  | 2000 | / | Inhibit the growth of S_180_ solid tumor and H22 liver cancer solid tumor and prolong the life of mice with ascites tumor | [38] |
|  | 2000 | / | Improve the memory damage induced by scopolamine, chloramphenicol and 40% ethanol on mice | [39] |
|  | 2000 | / | Reduce plasma lipids and arteriosclerosis in rats | [40] |
|  | 2005 | *α*-linolenic oil up to 22% | The toxicological and functional experiment showed that SWH oil was innocuous and harmless in Swiss mice and Wistar rats. | [41] |
|  | 2015 | Linoleic acid (**103**) | Antioxidant, antiglycemic, hypolipidemic activity | [7] |
| **Branch and leaves** | 1998 |  | 15-30 g (stem and branches) or 50-100 g (branches and leaves) boiled in water.  It was used for curing injuries from falls, fractures, contusions and strains, and rheumatism. | [42] |
|  | 2004 | Alkaloids, coumarins, terpenes, treterpenoid saponins, flavonoids, anthraquinones, tannins, etc. | Rat killing activity  At 20% content in feed of SWH stem and leaf, the killing to mice is 80%, and at 15% is 30% and at 10% is 10%, the safety experiment with the chicken expresses have no twice poisoning. | [43] |
| **Leaves** | 2011 |  | Ethyl acetate extract and ethanol extract has antibacterials effects to *Botrytis cinerea* Pers. Ex Fr. | [44] |
|  | 2012 | Quercetin, kaempferol | Butanol extract of SWH leaves significantly inhibited the paw edema on rat induced by 10% egg white. | [45] |
| **Whole herbs** | 2007 | Mineral elements, fibers, proteins, carbohydrates, vitamins, *β*-carotene and amino acids. (see attached tables ) | / | [66] |
| **Compound capsule** | 2009 | *Sambucus williamsii* Hance (stem), Drynaria fortune (Kunze) J. Sm. (rhizoma), Ligustricum wallichii Franch. (rhizoma), Astragalus membranaceus (Fisch.) Bunge (radix) | Reduced torsion time, increased the capillary permeability in abdominal cavity and decreased whole blood viscosity in mice. | [20] |
|  | 2009 |  | Promoted fracture healing | [18] |
|  | 2010 |  | Increased the capillary permeability and decreased blood viscosity to exert the effects of promoting blood circulation, antioncotic, analgesia and anti-inflammatory | [21] |
|  | 2010 |  | Relieved pain, improved fracture healing and promoted callus formation | [19] |
| **SWH**  **formula** | 2010 | *Sambucus williamsii* Hance and other herbs | Therapy for osteoarthritis in clinic | [67] |
